# Accuracy of Glenoid Component Placement in Total Shoulder Arthroplasty and Its Effect on Clinical and Radiological Outcome in a Retrospective, Longitudinal, Monocentric Open Study

**DOI:** 10.1371/journal.pone.0075791

**Published:** 2013-10-08

**Authors:** Thomas M. Gregory, Andrew Sankey, Bernard Augereau, Eric Vandenbussche, Andrew Amis, Roger Emery, Ulrich Hansen

**Affiliations:** 1 Department of Orthopaedic Surgery, APHP, European Hospital Georges Pompidou, Paris, France; 2 Mechanical Engineering, Imperial College, London, England; 3 Department of Orthopaedic Surgery, St Mary’s Hospital, London, England; 4 Division of Surgery, Imperial College, London, England; Cardiff University, United Kingdom

## Abstract

**Background:**

The success of Total Shoulder Arthroplasty (TSA) is believed to depend on the restoration of the natural anatomy of the joint and a key development has been the introduction of modular humeral components to more accurately restore the patient’s anatomy. However, there are no peer-reviewed studies that have reported the degree of glenoid component mal-position achieved in clinical practice and the clinical outcome of such mal-position. The main purpose of this study was to assess the accuracy of glenoid implant positioning during TSA and to relate it to the radiological (occurrence of radiolucent lines and osteolysis on CT) and clinical outcomes.

**Methods:**

68 TSAs were assessed with a mean follow-up of 38+/−27 months. The clinical evaluation consisted of measuring the mobility as well as of the Constant Score. The radiological evaluation was performed on CT-scans in which metal artefacts had been eliminated. From the CT-scans radiolucent lines and osteolysis were assessed. The positions of the glenoid and humeral components were also measured from the CT scans.

**Results:**

Four position glenoid component parameters were calculated The posterior version (6°±12°; mean ± SD), the superior tilt (12°±17°), the rotation of the implant relative to the scapular plane (3°±14°) and the off-set distance of the centre of the glenoid implant from the scapular plane (6±4 mm). An inferiorly inclined implant was found to be associated with higher levels of radiolucent lines while retroversion and non-neutral rotation were associated with a reduced range of motion.

**Conclusion:**

this study demonstrates that glenoid implants of anatomic TSA are poorly positioned and that this malposition has a direct effect on the clinical and radiological outcome. Thus, further developments in glenoid implantation techniques are required to enable the surgeon to achieve a desired implant position and outcome.

## Introduction

Anatomic Total Shoulder Arthroplasty (TSA) is an effective treatment for shoulder arthritis. However, a meta-analysis [1] pointed out that loosening rates are high and that glenoid component loosening is the main complication after TSA. There is a consensus perception that the goal and success of TSA depend on the restoration of the natural anatomy of the joint and major developments in the past decade have included the introduction of modular humeral components to more accurately restore the patient’s anatomy [2].

Implanting the glenoid component in an anatomical position is a challenging procedure and this difficulty may explain the high rates of glenoid component loosening. There are three main reasons why the accurate positioning of the glenoid component is difficult. Firstly, the bone stock of the native glenoid is very limited and often further reduced by arthritic wear and erosion. Secondly, the anatomical position is poorly understood and shows great patient-specific variability [3], [4]. Hence, surgeons do not know what position they should aim for and instead aim for a ‘standard’ position of so-called neutral orientation of the glenoid component with respect to the scapula. Thirdly, there are no reliable landmarks to determine the position of the blade of the scapula intra-operatively, which is essential for accurate placement of the glenoid component in the standard position. Because of the lack of scapular landmarks, the surgeon may be guided by the perceived position and orientation of the exposed glenoid bone surface. However, due to the patient-specific variability, as well as erosion and wear, such a position is likely to deviate from the standard position.

Previous computational and in-vitro studies have indicated the importance of glenoid component position by demonstrating that mal-positioned implants caused abnormal loading of the glenoid component and a resulting higher risk of mechanical failure [5], [6], [7], [8]. Hence, in clinical practice the surgeon will often attempt to insert the implant in a ‘standard’ position of neutral (0°) version, inclination and rotation. However, there are no peer-reviewed studies that have reported the degree of mal-position achieved in clinical practice and the clinical outcome of such mal-position.

The purposes of this study were to assess the accuracy and variability of glenoid implant positioning during TSA and to relate it to the radiological (occurrence of radiolucent lines and osteolysis on CT) and clinical outcomes. The hypothesis, based on the literature reviewed, was that the clinical and radiological outcomes would correlate with the accuracy of glenoid component position.

## Materials and Methods

Post-operative CT-scanning of TSAs is part of a routine patient care in our institution and therefore patient consent was not needed. The study was approved by the ethics review committee “Centre d’Éthique Clinique, CERES” on November 2012. The committee testified that the study was in accordance with the scientific principles generally accepted and with the ethical standards of research and waived the need for patient consent.

We reviewed a series of 68 consecutive anatomic TSA from a single centre, performed by 1 senior consultant shoulder surgeon between 2002 and 2008. The series included 59 female and 9 male patients; 39 patients were right handed and 9 were left handed. All glenoid implants of the series were keeled cemented polyethylene implants. Among them, 45 were Neer II (Smith and Nephew), and 23 Ulys (Ceraver). Other data relating to this patient series is shown in Table 1.

**Table 1:** Patient data and measured implant position parameters.

	Average	SD	Range
Glenoid version (°)	8	9	[−17, 32]
Glenoid inclination (°)	12	13	[−21, 50]
Glenoid rotation (°)	7	14	[−30, 46]
Glenoid Off-set (mm)	4	3	[−7, 15]
Humeral position (mm)	1	3	[−11, 8]
Humeral retroversion (°)	41	12	[11, 62]
Glenohumeral misalignment (mm)	0	1	[−3, 4]
Age (years)	69	10.5	[38, 88]
Radiographic follow-up (months)	38	27	[12, 159]
Preoperative status of rotator cuff	8 partial thickness supraspinatus tears, 10 full thickness tears (4 trans osseous reattachments)		
Shoulder etiology	53 primary OA, 7 RA, 6 post-traumatic OA,		
(ostearthritis OA, rheumatoid arthritis RA)	2 post instability OA		

**Figure 1:**
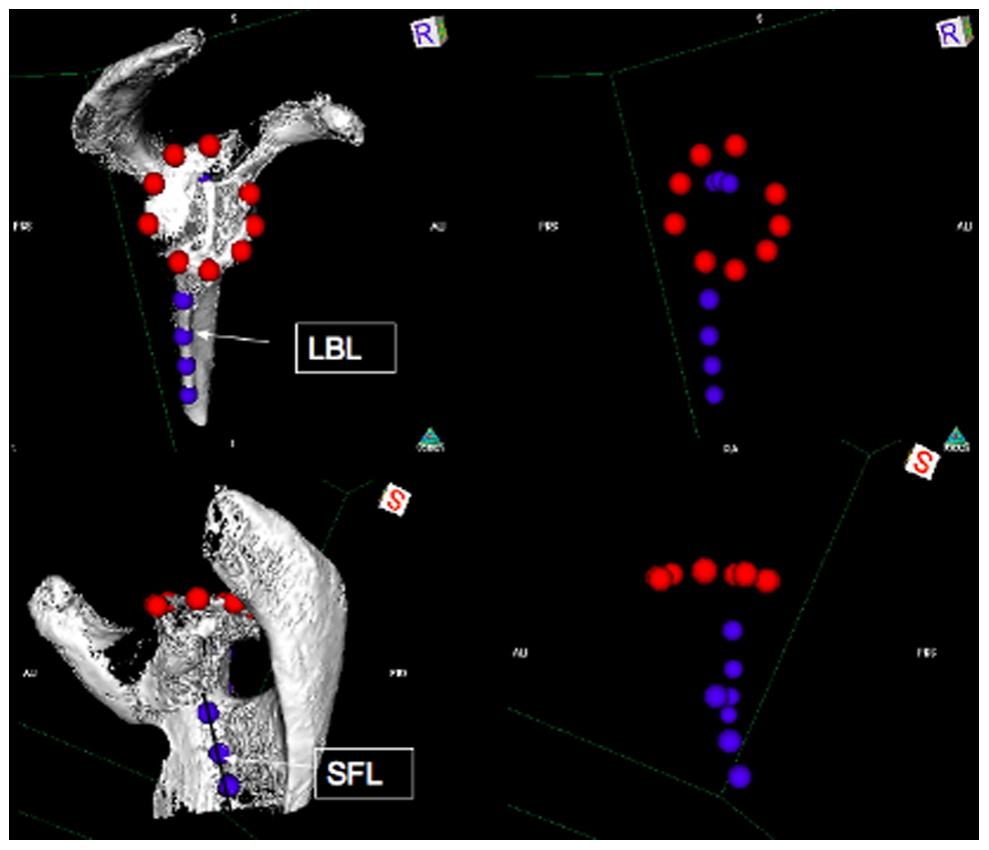
Digitised landmarks used to define the relative position of the glenoid bone relative to the scapular blade. On the left is shown the digitized points used to establish the glenoid surface plane and the scapula plane. On the right is shown the digitized points outlining the glenoid surface and scapula. Also shown are the lines labelled LBL (Lateral Border Line) and SFL (Supraspinatus Fossa Line) used to determine the scapula plane as well as the inclination.

All patients were assessed with a CT-scan some time after the operation (the radiological follow-up). The average follow-up is shown in Table 1. A CT-scan protocol that minimized artefacts produced by the metallic humeral component allowing excellent visualization of the glenoid component fixation was used [8], [9]. The raw CT data were analyzed with computer software OsiriX® [10], [11]. The scapulae were reconstructed in 3D and digitized reference points were positioned along the lateral border of the scapula, approximately a line (LBL), as well as along the deepest part of the near-linear Supraspinous Fossa Line (SFL) (Figure 1). These two sets of points, effectively two lines, determine the plane of the blade of the scapula. Additional points were digitized along the edge of the subchondral bone of the glenoid, in order to evaluate the position of the glenoid component relative to the plane of the scapula (Figure 1). Using the digitized points, another software (3D-Reshaper®) calculated the best approximation to a flat plane of the scapula and of the glenoid surface, as well as the relative position and orientation of these two planes [12]. Based on these CT scan measurements the parameters describing the implant position were determined.

**Figure 2:**
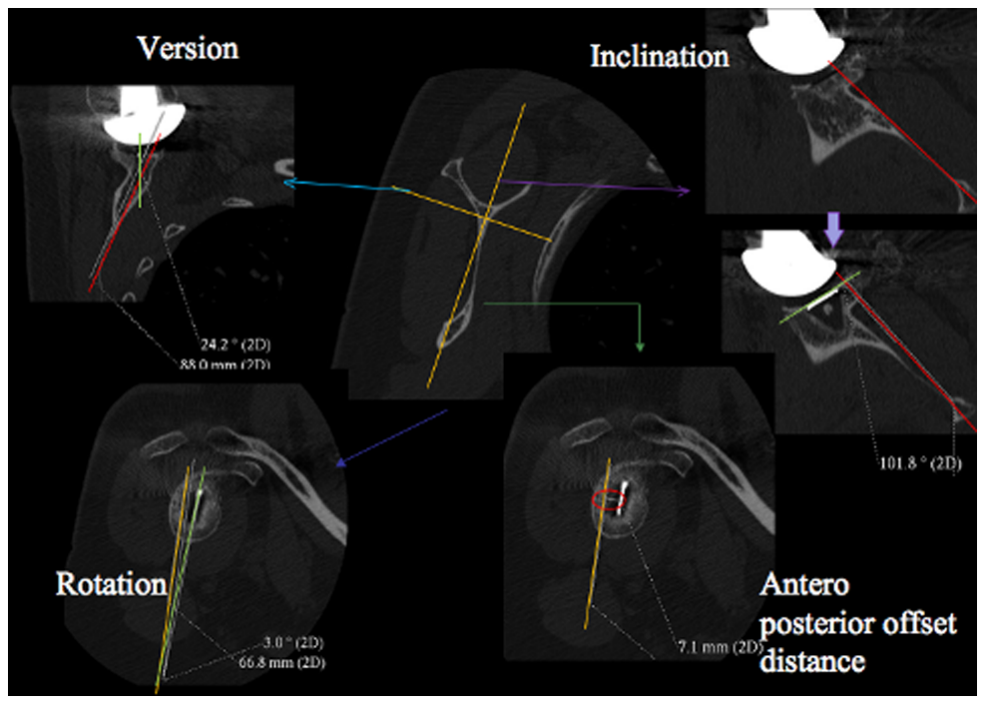
Definition of the four glenoid component positioning parameters. : version (top left) is the angle between the green line (i.e. the orientation of the glenoid component surface) and the red line (i.e. the orientation of the scapula plane); inclination (top right) was measured from the orientation of green line (i.e. the superior-inferior line of the glenoid component) and the red line (i.e. the orientation of the supraspinatus fossa line (SFL)); rotation (bottom left) is the angle between the orange line (i.e. the orientation of the scapula plane) and the green line (i.e. the superior-inferior line of the glenoid component); off-set distance(bottom right) was measured as the shortest distance between the white marker in the CT image (i.e. the centre of the glenoid) and the orange line (i.e. the orientation of the scapula plane). The orange lines in the middle image indicate the orientation of the scapula blade used.

Four parameters of the glenoid component position were studied: the version, the inclination, the rotation and the anterior-posterior off-set distance from the scapular plane (Figure 2). Version was defined as the angle between the plane of the glenoid component surface and the plane of the scapula and was scored so that retroversion was positive and anteversion negative. The inclination was defined as the angle between the superior-inferior line of the glenoid component (i.e. the line that connects the most superior point of the glenoid surface to the most inferior point) and the supraspinous fossa line (SFL) and was scored so that superior inclination was positive and inferior inclination negative. However, in this work the superior-inferior line was easily estimated by measuring the orientation of the radiographic marker imbedded in the glenoid implant (Figure 2, top right). Rotation was defined as the angle between the scapula plane and the superior-inferior line of the glenoid component as described above. Clockwise rotation was considered positive for a right shoulder and anticlockwise rotation was considered positive for a left shoulder. The off-set was measured as the shortest distance between the centre of the glenoid and the scapula plane and was always anterior to the scapular plane.

**Figure 3:**
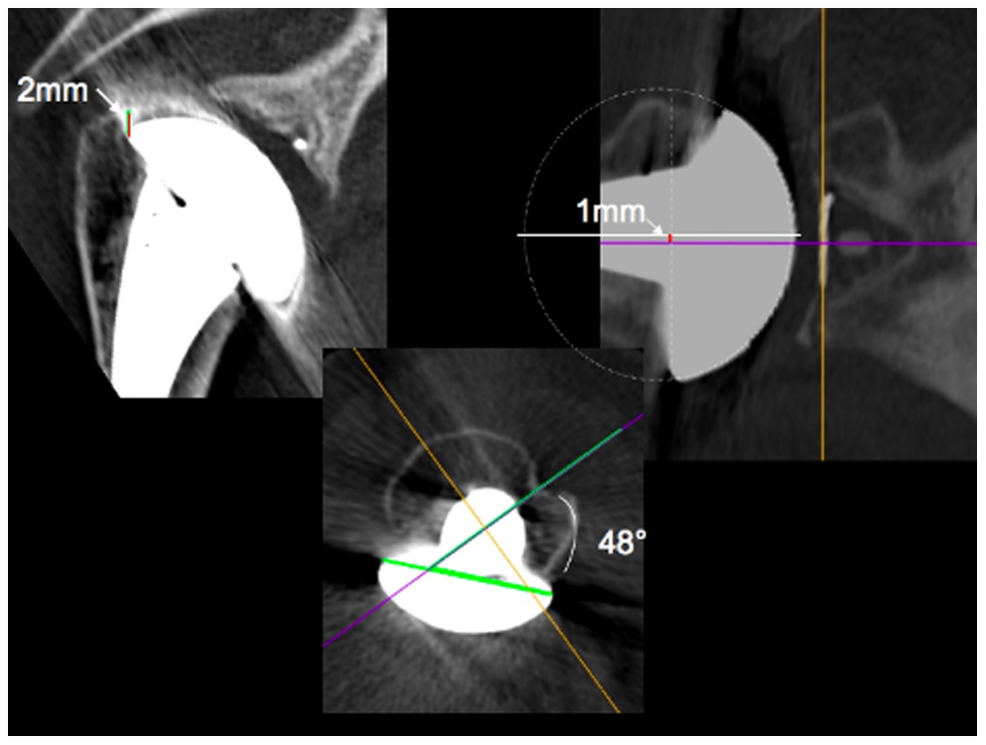
Definition of the three the humeral head component positioning parameters. : humeral head position (left) relative to the greater tuberosity indicated by the black line; retroversion (middle) of the humeral component was measured as the angle between the green line (i.e. the diametrical line across the humeral head component) and the purple line (i.e. the line that joins the bicipital groove to the centre of the femoral shaft); Glenohumeral misalignment (right) was measured as the distance between the white line (i.e. the centre of the humeral head) and the purple line (i.e. the centre of the glenoid component).

Three parameters relating to the humeral head position [13], [14] and illustrated in Figure 3 were also studied: humeral position (i.e. humeral head position relative to the greater tuberosity) which was scored as positive if the humeral head position was above the greater tuberosity; humeral retroversion (i.e. orientation of the humeral component relative to a line joining the bicipital groove to the centre of the femoral shaft); glenohumeral misalignment (i.e. the relative position of the centre of the humeral head relative to the centre of the glenoid component) which was given a positive value if the humeral head centre was superior to the glenoid component centre.

All patient medical reports contained a clinical assessment at one year after the operation (one-year follow-up). The clinical assessment included ranges of shoulder motions (i.e. forward flexion, abduction and external rotation) and a Constant Score. This information constituted the clinical results used in this study. A high range of motion indicates a well functioning shoulder while the Constant Score [15] assesses the functioning of a patient’s shoulder based on several other components. The value of the score can range from 0 to 100, 0 being poor and 100 the score of a very well functioning shoulder. The components of the score consist of 15 points assigned based on pain, 20 points based on subjective assessment of ability to carry out daily functions, 40 points assessed on measured ability to move the arm and 25 points based on measured strength of the shoulder.

**Figure 4:**
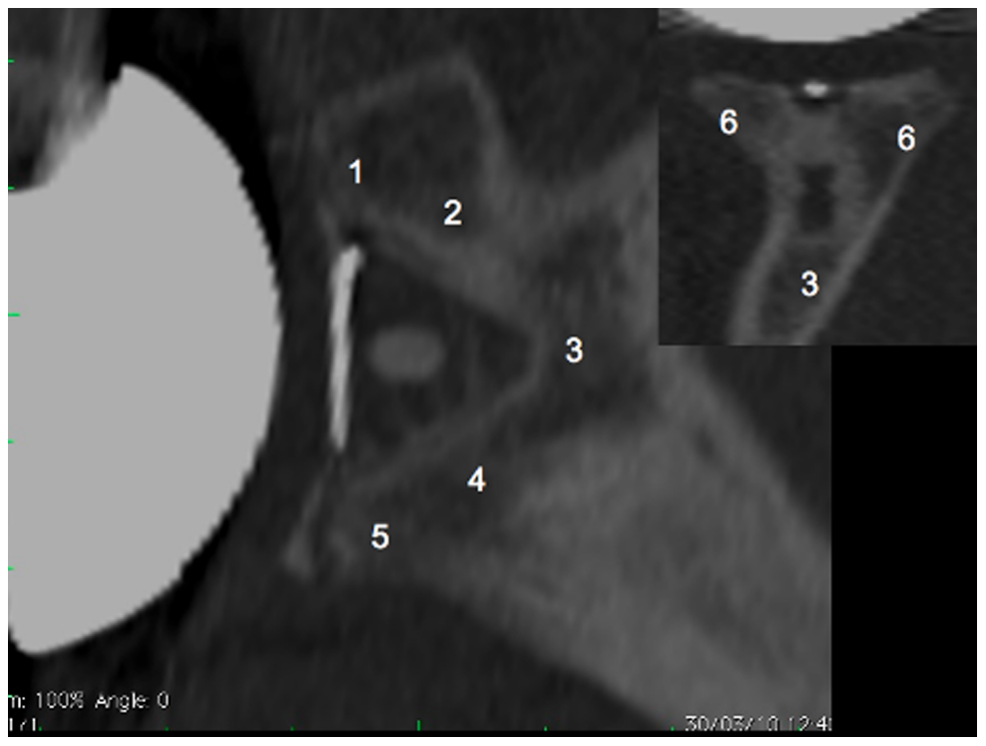
Sagittal and coronal views of the glenoid bone including the implant. The numbers indicate the six zones used in the Mole Score to assess the level of radiolucent lines in the fixation of the glenoid component. Zone 1: fixation area of the superior part of the glenoid component base plate; Zone 2: fixation area of the superior part of the keel; Zone 3: fixation area of the tip of the keel; Zone 4: fixation area of the inferior part of the keel; Zone 5: fixation area of the inferior part of the glenoid component base plate; Zone 6: fixation area of the central part of the glenoid component base plate. Each zone is scored between 0 and 3 points according to the level of radiolucent lines observed and the Mole Score is the sum of these scores.

**Figure 5:**
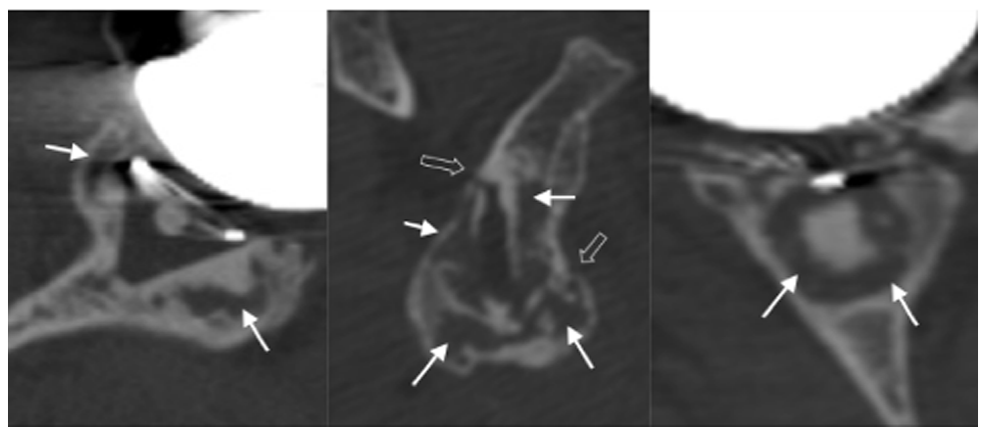
Osteolysis (indicated by the white arrows) and cortical disruption (indicated by the double arrows). Sagittal plane of the glenoid implant (left); axial plane of the implant (middle) and in the coronal plane of the implant (right).

In addition to allowing determination of the implant position, the CT scans at the latest follow-up also allowed assessment of the levels of radiolucent lines and presence of osteolysis. These radiological assessments were done by both a senior consultant shoulder surgeon as well as a musculoskeletal radiologist through a mutual analysis. Radiolucent lines were ranked according to the Mole Scoring system [16]. The Mole Score quantifies the level of radiolucent lines in the fixation of the glenoid component and takes a value between zero and 18. A Mole Score of zero indicates no radiolucent lines, i.e. ‘good’ while a score of 18 indicates an extensive level of radiolucent lines, i.e. ‘bad’. The method divides the region around the glenoid component into the 6 zones indicated in Figure 4. Each zone is then scored from 0 to 3: ‘0’ if there are no radiolucent lines in the zone, ‘1’ if the radiolucent line is less than 1 mm thick, ‘2’ if the radiolucent line is between 1 to 2 mm thick and ‘3’ if the radiolucent line is more than 2 mm thick. Osteolysis was ranked according to a 4 criteria scale: no osteolysis, osteolysis restricted to one or more of the glenoid component fixation areas, major osteolysis involving the whole glenoid component fixation without cortical disruption, and major osteolysis involving the whole glenoid component fixation with cortical disruption (Figure 5).

### Statistical Evaluation

Statistical analysis software SPSS 10.0 (SPSS, Chicago, Illinois) was used to determine relationships between variables. Significance was set at p<0.05. The version, the inclination, the rotation and the off-set distance of the glenoid component; the position and the retroversion of the humeral head; the glenohumeral misalignment; the age of the patient at the time of surgery and the time between surgery and the radiographic follow-up session; the preoperative status of the rotator cuff and the shoulder etiology were all analyzed in relation to forward flexion, abduction, external rotation, Constant Score and the Mole Score using ordinary least squares techniques, with a progressive withdrawal of the non significant values. Square and cube exponents were introduced for the positioning parameters in order to take into account non linear effects.

## Results

### Implant Positioning Parameters

The measured implant position parameters are shown in Table 1. The average glenoid version was 8° but the standard deviation of 9° and range from 17° of anteversion to 32° of retroversion show that there was great variation in glenoid component version. Table 1 also shows large variability of the other implant position parameters.

### Clinical Outcome at 12 Months Follow-up

**Figure 6:**
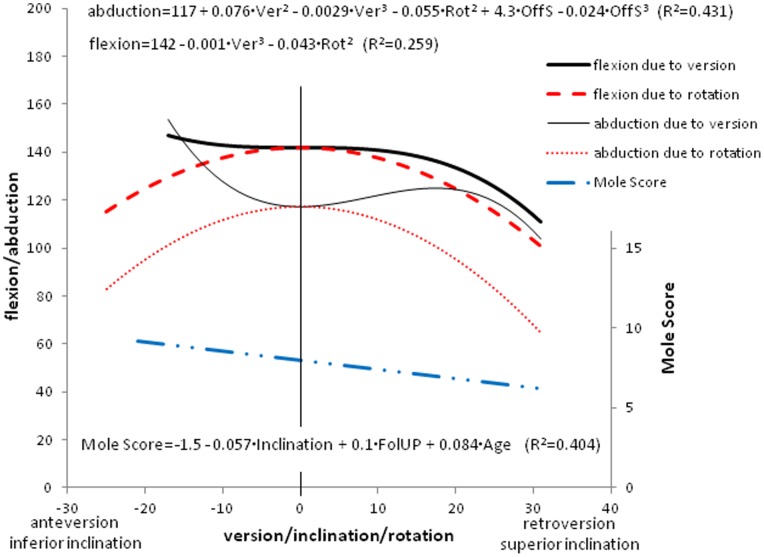
Regression equations of identified statistically significant relationships between patient and implant position parameters and clinical and radiographic outcomes. Ver: version, Rot: rotation, OffS: off-set, FolUP: follow-up. The graphs illustrate these effects of implant position parameters on clinically measured ranges of shoulder motion and Mole Score, respectively.

The clinical results are reported in Table 2 and the results of the statistical analysis are reported in Table 3. Note that most relationships were found to be statistically non-significant. Only those that were significant were further analysed leading to the regression models and results shown in Figure 6. Shoulder forward flexion and abduction were significantly negatively correlated with the version and rotation of the glenoid component. Figure 6 shows the effects of version and rotation on flexion and abduction, as determined by the regression equations. Figure 6 indicates that more than approximately 20° of retroversion will result in reduced flexion as well as abduction while anteversion seems to improve abduction. Figure 6 also shows that neutral rotation results in the highest range of both flexion and abduction. Similar analyses showed that an off-set distance of 8 mm resulted in the highest range of abduction.

**Table 2:** Outcome of surgery: clinical and radiological results.

	Average	Standard deviation	Range
Forward flexion (°)	130	29	[40, 180]
Abduction (°)	125	31	[25, 180]
External Rotation	28	19	[0, 150]
Constant Score	69	19	[7, 90]
Mole Score	7.5	4.2	[0, 15]

**Table 3:** P-values from analysis investigating existence of statistical significant relationships between patient/implant position parameters(Table 1) and clinical/radiological results(Table 2).

Outcome measure	Glenoid component				Humeral component		Glenohumeral misalignment	Followup	Age	Rotator cuff status	Shoulder etiology
	version	inclination	rotation	off-set	position	version					
Forward flexion	0.017* ++	0.206	0.004* +	0.293	0.769	0.185	0.05	0.239	0.925	0.252	0.461
Abduction	0.048* 0.015* ++	0.814	<0.001* +	0.011*	0.514	0.112	0.198	0.7	0.840	0.402	0.920
External rotation	0.613	0.975	0.713	0.921	0.401	0.889	0.250	0.898	0.683	0.808	0.918
Constant Score	0.887	0.744	0.903	0.903	0.524	0.507	0.858	0.723	0.538	0.802	0.973
Mole Score	0.647	0.038	0.787	0.974	0.881	0.811	0.648	<0.001*	<0.05*	0.413	0.610

* Indicates statistical significance.

+ square relationship with independent variable.

++ cubic relationship with independent variable.

### Radiological Outcome at the Latest Follow-up Evaluation

The result of the Mole Score assessments is reported in Table 2. The Mole Score was significantly correlated with glenoid component inclination, patient follow-up and age (Table 3). The Mole lucency score was significantly negatively correlated with the inclination of the glenoid component, and positively correlated with the age of the patient and the follow-up, hence as their radiolucency scores were high, inferiorly inclined implants performed less well (Figure 6).

21 patients (36%) had no osteolysis around the glenoid component, 17 (29%) had osteolysis restricted to one or more of the glenoid component fixation areas, 14 (24%) had major osteolysis involving the whole glenoid fixation without cortical disruption, and 6 (10%) had major osteolysis involving the whole glenoid component fixation with cortical disruption.

## Discussion

This study demonstrates that the glenoid component of so-called anatomic TSA is not positioned in a standard fashion relative to the scapula plane, and that (mal)position affects the prevalence of radiolucencies as well as the clinical outcome.

A unique feature of this work is the particular CT protocol used. This protocol was developed in previous work [2], [8], [9] and allows radiolucent lines to be analyzed more accurately than previously possible. Furthermore, CT techniques are more accurate in determining glenoid component position than the standard axial radiographs [17] used in previous studies on glenoid positioning. These two characteristics of the CT method have enabled this study to report the clinically observed association between glenoid component position and radiolucent lines, which has not been done before.

It is a limitation of the study that all replacement surgery in this series was performed by only one surgeon and, strictly, it represents the accuracy achieved by just this surgeon. Never-the-less, the fact that even in the hands of one very experienced surgeon, in a series involving 68 anatomical TSA, the position was very variable and the standard position only rarely achieved emphasizes the problem of glenoid component positioning during surgery, and the reported values are consistent with data reported by Gregory et al [12] in a previous study of 29 cases.

### Achieved Position

Very few studies have reported on the accuracy of glenoid component positioning achieved during surgery. Moska et al. [18] presented data from 133 patients showing that 82% of glenoid components were positioned within neutral and 20° of retroversion. Nyffeler et al. [17] reported from a series of 25 glenoid implants that the version ranged from 16° of anteversion to 23° of retroversion with an average of 2° of anteversion. From a series of 10 patients, Kircher et al. [19] reported the version to range from neutral to 19° of retroversion (11°±7°; mean ± SD). These previous data showing a large spread around a somewhat retroverted position are broadly consistent with the findings of the present study. These results reflect the reported position of the native non-arthritic glenoid [4] and/or may reflect that surgeons are guided by the orientation of the native glenoid surface which is often eroded posteriorly in candidates for TSA [12].

### Effect of Implants Positioning on Outcomes

Fanta et al. [14] also analysed the impact of humeral head position and glenohumeral misalignment on clinical outcomes. They concluded that an excessively high humeral component position and glenohumeral misalignment was associated with unsatisfactory results. Our results do not support these findings. However, in the study of Fanta et al., these associations were related to humeral head positions greater than 10 mm and misalignments greater than approximately 12 mm. In comparison, the humeral head positions and misalignments found in our study rarely, if ever, reached such large values (Table 2). Thus the humeral component in our study may simply have been too well placed to capture the effects observed by Fanta et al. It is also worth noting that we found the effect of glenohumeral alignment on forward flexion to be very close to statistical significance (Table 3).

Our study analyses the impact of the glenoid component placement on clinical and radiological outcome. To our knowledge, only Moska et al. [15] analysed the influence of glenoid component version on clinical results, and our paper is the first to analyse the effect of the others glenoid component positioning parameters, namely the inclination, the rotation and the anterior offset distance.

Moska et al. [18] reported that glenoid components implanted in version ranging from neutral to 20° of retroversion were associated with excellent patient satisfaction whereas version outside this range was associated with patient dissatisfaction. The importance of version was confirmed in our study as we found that version affected both abduction and flexion, which may be related to patient satisfaction. Consistent with Moska et al., we also found that flexion and abduction deteriorated beyond 20° of retroversion (Figure 6). However, in the current study anteversion seemed to result in an improved range of abduction.

Our analysis has so far indicated that any position of anteversion was well tolerated. However, the number of patients in our study with noticeably anteverted implants (more than 5° of anteversion) was relatively small (n = 7) and further studies including more anteverted implants would be necessary before making conclusive decisions. Considering the result of Moska et al. [18], a more cautious recommendation is to aim for a glenoid component position of neutral version. In addition, our results pointed out that neutral rotation and an anterior offset distance of 8 mm were associated with optimal clinical results.

Several in-vitro studies indicated that superior inclination will lead to superior migration of the humeral head which in turn will cause impingement and rotator cuff tears and ultimately poor outcome [20], [21], [22]. In our clinical study, inclination was not found to have any significant effect on either the ranges of motion or the Constant score and hence do not support these suggestions of earlier papers. Terrier et al. [23] suggested that, whereas superior inclination might be expected to be worse for the reasons of impingement and cuff tears, it may on-the-other-hand result in better conditions for the fixation. The results of our clinical study do support this suggestion as we found higher rates of radiolucent lines for inferiorly inclined implants.

In-vitro studies [5], [6], [7], [20] have investigated the effect of glenoid implant position on the forces imparted on the implant fixation. They all concluded that non-neutral version and inclination will lead to more eccentric loading of the implant and, hence, to an increased risk of fixation failure. Our clinical results do not support these suggestions as we found no correlation between version and radiolucent lines. Indeed we found superior inclination associated with low levels of radiolucent lines. The explanation for this inconsistency may be that during surgery surgeons will often try to correct mal-position but in doing so jeopardize the osseous fixation [12]. The effect of corrective procedures was not taken into account in the earlier biomechanical studies and may explain the inconsistency with our results.

In regards to superior inclination, most of our data points are for superior inclinations of less than 20° and our conclusions should probably not be extended beyond this level of inclination. Unexpectedly, we found no statistical significant relationships between neither the state of the rotator cuff nor the shoulder etiology and clinical/radiological results. This may have been related to an insufficient number of patients (only 10 full thickness cuff tears and only 7 rheumatoids) and that our analysis dropped parameters that were associated with P-values ≥0.05 from the final regression model relationships shown in Figure 6. From Table 3 it can be seen that several parameters were associated with P-values close to 0.05 and as in any statistical significance analysis this indicates that there is a 5% risk that our findings of non-significance were wrong.

### Differences in Methodologies Compared with Other Studies

When comparing our results with the above studies as well as with studies reporting the orientation of the native glenoid there are differences in methodology that may cause differences in quantitative values. In this study the reference plane of the scapula was based on the lateral border of the scapula and the deepest part of the supraspinous fossa. This differs from the definition used in other studies [3], [4], [14], where the plane was defined according to three points: the centre point of the native glenoid surface, the point where the scapular spine meets the medial border and the inferior angle of the scapula. For the purpose of limiting the radiation exposure, the acquisition field of the CT-scans in our study was restricted to the part of the scapula nearest to the glenoid region. Therefore, two of the necessary reference points used by the previous studies were not available and we could not use these previous methods. For similar reasons we defined inclination relative to the near-horizontal line of the supraspinous fossa (SFL) while others [3] have defined inclination with respect to the line from the centre of the glenoid surface to the vertebral border of the scapula. The SFL is approximately 5° superiorly inclined to this line.

Barring these practical difficulties if using previous definitions of the scapular plane and the reference line for inclination, there are also advantages of the method used in this study. Amadi et al. [24] showed that a scapular plane based on the same two lines as in this study provided the most consistent definition of landmark lines and of the scapular blade irrespective of arthritic and other osseous changes. As the shoulder joint in TSA often exhibits osseous changes this is an important advantage.

## Conclusion

This study found that malposition of the glenoid implant has a direct effect on the clinical and radiological outcome. We recommend implanting the glenoid component in neutral rotation, neutral to slightly retroverted and neutral to 20° of superior inclination, but further studies are needed to establish consensus on the optimum level of version and inclination. However, this study also demonstrates that glenoid implants are poorly positioned even by very experienced specialized surgeons. This is due to the inherent difficulty of the operation and further developments in glenoid implantation techniques are required to enable the surgeon to achieve a desired implant position.

## References

[1] Bohsali KI, Wirth MA, Rockwood CA Jr (2006) Complications of total shoulder arthroplasty. J Bone Joint Surg Am 88: 2279–2292. 17015609 10.2106/JBJS.F.00125

[2] Gregory T, Hansen U, Emery RJ, Augereau B, Amis AA (2007) Developments in shoulder arthroplasty. Proc Inst Mech Eng H 221: 87–96. 17315772 10.1243/09544119JEIM167

[3] Friedman RJ, Hawthorne KB, Genez BM (1992) The use of computerized tomography in the measurement of glenoid version. J Bone Joint Surg Am 74: 1032–1037. 1522089

[4] Churchill RS, Brems JJ, Kotschi H (2001) Glenoid size, inclination, and version: an anatomic study. J Shoulder Elbow Surg 10: 327–332. 11517362 10.1067/mse.2001.115269

[5] Hopkins AR, Hansen UN, Amis AA, Emery R (2004) The effects of glenoid component alignment variations on cement mantle stresses in total shoulder arthroplasty. J Shoulder Elbow Surg 13: 668–675. 15570237 10.1016/S1058274604001399

[6] Oosterom R, Rozing PM, Bersee HE (2004) Effect of glenoid component inclination on its fixation and humeral head subluxation in total shoulder arthroplasty. Clin Biomech (Bristol, Avon) 19: 1000–1008. 10.1016/j.clinbiomech.2004.07.001 15531049

[7] Nyffeler RW, Sheikh R, Atkinson TS, Jacob HA, Favre P, et al (2006) Effects of glenoid component version on humeral head displacement and joint reaction forces: an experimental study. J Shoulder Elbow Surg 15: 625–629. 16979061 10.1016/j.jse.2005.09.016

[8] Gregory T, Hansen U, Taillieu F, Baring T, Brassart N, et al.. (2009) Glenoid loosening after total shoulder arthroplasty: An in vitro CT-scan study. J Orthop Res. 10.1002/jor.20912 19472376

[9] Gregory T, Taillieu F, Baring T, Vandenbussche E, Hansen UN, et al.. (2007) A new CT-scan protocol for the in-vivo analysis of glenoid failure after total shoulder arthroplasty. 10th International Congress on Shoulder Surgery Salvador, Bahia, Brasil.

[10] Rosset A, Spadola L, Ratib O (2004) OsiriX: an open-source software for navigating in multidimensional DICOM images. J Digit Imaging 17: 205–216. 15534753 10.1007/s10278-004-1014-6 PMC3046608

[11] Rosset C, Rosset A, Ratib O (2005) General consumer communication tools for improved image management and communication in medicine. J Digit Imaging 18: 270–279. 15988626 10.1007/s10278-005-6703-2 PMC3046724

[12] Gregory T, Hansen U, Emery R, Amis AA, Mutchler C, et al (2012) Total shoulder arthroplasty does not correct the orientation of the eroded glenoid. Acta Orthop 83: 529–535. 23083436 10.3109/17453674.2012.733916 PMC3488182

[13] Franta AK, Lenters TR, Mounce D, Neradilek B, Matsen FA 3rd (2007) The complex characteristics of 282 unsatisfactory shoulder arthroplasties. J Shoulder Elbow Surg 16: 555–562. 17509905 10.1016/j.jse.2006.11.004

[14] Hempfing A, Leunig M, Ballmer FT, Hertel R (2001) Surgical landmarks to determine humeral head retrotorsion for hemiarthroplasty in fractures. J Shoulder Elbow Surg 10: 460–463. 11641704 10.1067/mse.2001.117127

[15] Franklin JL, Barrett WP, Jackins SE, Matsen FA 3rd (1988) Glenoid loosening in total shoulder arthroplasty. Association with rotator cuff deficiency. J Arthroplasty 3: 39–46. 3361319 10.1016/s0883-5403(88)80051-2

[16] Mole DRO, Riand N, Levigne C, Walch G (1999) Cemented glenoid components: results in osteoarthritis and rheumatoid arthritis. In: Walch G BP, editor. Shoulder arthroplasty. Berlin: Springer. 163–171.

[17] Nyffeler RW, Jost B, Pfirrmann CW, Gerber C (2003) Measurement of glenoid version: conventional radiographs versus computed tomography scans. J Shoulder Elbow Surg 12: 493–496. 14564275 10.1016/s1058-2746(03)00181-2

[18] Moska MJ, Duckworth D, Matsen FA (1998) Contrasting the position of prosthetic joint surfaces in sucessful and failed shoulder arthroplasties. 7th International Congress on Shoulder Surgery. Sydney, Australia. 5–8.

[19] Kircher J, Wiedemann M, Magosch P, Lichtennberg S, Habermeyer P (2009) Improved accuracy of glenoid positioning in total shoulder arthroplasty with intraoperative navigation: a prospective-randomized clinical study. Journal of Shoulder and Elbow Surgery 18: 515–520. 19559369 10.1016/j.jse.2009.03.014

[20] Favre P, Mor B, Snedeker JG, Gerber C (2008) Influence of component positioning on impingement in conventional total shoulder arthroplasty. Clinical Biomechanics 23: 175–183. 17983693 10.1016/j.clinbiomech.2007.09.009

[21] Iannotti JP, Spencer EE, Winter U, Deffenbiugh D, Williams G (2005) Prosthetic positioning in total shoulder arthroplasty. Journal of Shoulder and Elbow Surgery 12. 10.1016/j.jse.2004.09.026 15726070

[22] Konrad GG, Markmiller M, Jolly JT, Ruter AE, Sudkamp NP, et al (2006) Decreasing glenoid inclination improves function in shoulders with simulated massive rotator cuff tears. Clinical Biomechanics 21: 942–949. 16781027 10.1016/j.clinbiomech.2006.04.013

[23] Terrier A, Merlini F, Pioletti D, Farron A (2009) Total shoulder arthroplasty: downwards inclination of the glenoid component to balance supraspinatus deficiency. Jounral of Shoulder and Elbow Surgery 18: 515–520. 10.1016/j.jse.2008.11.008 19243979

[24] Amadi HO, Hansen UN, Wallace AL, Bull AM (2008) A scapular coordinate frame for clinical and kinematic analyses. J Biomech 41: 2144–2149. 18555258 10.1016/j.jbiomech.2008.04.028

